# Comparison of Anthropometric and Physical Performance Profiles in Elite Judo and Jiu-Jitsu Athletes

**DOI:** 10.3390/sports14050207

**Published:** 2026-05-18

**Authors:** Artur Avelino Birk Preissler, Filipe Manuel Clemente, Marcela Zimmermann Casal, Rui Miguel Silva, Ana Filipa Silva, João Vitor Silveira, Pedro Schons

**Affiliations:** 1 Faculdade SOGIPA, Porto Alegre 90240-485, RS, Brazilpedroschons@hotmail.com (P.S.); 2 School of Physical Education, Physiotherapy and Dance, Federal University of Rio Grande do Sul, Porto Alegre 90690-200, RS, Brazil; 3 Department of Biomechanics and Sport Engineering, Gdansk University of Physical Education and Sport, 80-336 Gdańsk, Poland; filipe.clemente5@gmail.com; 4 Applied Research Institute (i2A), Polytechnic University of Coimbra, 3045-093 Coimbra, Portugal; 5 The Sport Physical Activity and Health Research & Innovation Center, 3045-093 Coimbra, Portugal; 6 Escola Superior de Desporto e Lazer, Instituto Politécnico de Viana do Castelo, 4960-320 Viana do Castelo, Portugal; 7 The Sport Physical Activity and Health Research & Innovation Center, 4960-320 Viana do Castelo, Portugal; 8 Faculty of Sport Sciences and Physical Education, University of Coimbra, 3040-256 Coimbra, Portugal

**Keywords:** judo, Brazilian jiu-jitsu, combat sports, anthropometric profile, strength, power, elite athletes

## Abstract

Combat-sport performance depends on the interaction between technical skills and physical capacities, yet direct comparisons between grappling disciplines remain limited. The aim of this study was to compare the anthropometric profile and physical performance of elite judo and jiu-jitsu athletes. This cross-sectional study included 25 elite male athletes (judo—*n* = 12; jiu-jitsu—*n* = 13) assessed during a preparatory training phase. Anthropometric measures included age, training experience, height, and body mass, while physical performance was evaluated using dominant and non-dominant handgrip strength, squat jump (SJ), countermovement jump (CMJ), medicine ball throw with and without countermovement, and dynamic and isometric judogi-grip pull-up tests. Between-group comparisons were performed using independent sample tests, with effect sizes (ES) calculated. Judo athletes had greater training experience (13.25 ± 2.73 vs. 7.85 ± 4.36 years; *p* = 0.001; ES = 1.472) and higher SJ performance (38.71 ± 6.69 vs. 33.82 ± 4.74 cm; *p* = 0.045; ES = 0.850) compared to jiu-jitsu athletes. No significant between-group differences were observed for the remaining variables (*p* > 0.05). These findings indicate that no statistically significant differences were detected in most anthropometric and physical performance variables between elite judo and jiu-jitsu athletes, and the initially higher squat jump performance observed in judo athletes was no longer statistically significant after adjustment for training experience.

## 1. Introduction

Combat-sport performance in grappling disciplines such as judo and Brazilian jiu-jitsu emerges from the interaction between technical skill and physical qualities such as maximal strength, neuromuscular power, anaerobic capacity, and body composition. Grappling disciplines place especially high demands on force production, isometric grip endurance, and intermittent high-intensity efforts interspersed with brief pauses [1,2,3]. Because these traits influence training prescription, talent identification, and interpretation of sport-specific tests, defining the physical profile of each modality is a relevant applied problem for coaches and practitioners [1,2]. However, direct comparisons between elite judo and Brazilian jiu-jitsu athletes under standardized conditions remain limited, restricting understanding of sport-specific adaptations.

Judo and Brazilian jiu-jitsu are closely related grappling sports, but their competitive structures are not identical [4,5,6]. Contemporary judo competition is strongly influenced by standing exchange, explosive throwing actions, and rule-driven variations in bout duration, typically characterized by shorter matches and intermittent high-intensity effort pause patterns, whereas Brazilian jiu-jitsu typically includes longer ground exchanges and a distinct effort–pause structure centered on positional control and submission, often involving longer match durations and more prolonged effort phases [5,6]. Moreover, athletes frequently transition between judo and Brazilian jiu-jitsu for competitive or training purposes, seeking technical and physical adaptations that may transfer between modalities. These technical and temporal distinctions suggest that some physical qualities may converge across disciplines, such as grip-related strength and endurance, whereas others may adapt in a modality-specific manner, particularly lower limb explosive power and movement-specific neuromuscular demands [1,4].

In judo, studies and reviews consistently describe high-level athletes as lean, powerful, and dependent on upper- and lower-body strength qualities that contribute to sport-specific performance [7,8,9]. In Brazilian jiu-jitsu, systematic and observational evidence likewise indicates low body fat and a predominantly mesomorphic profile, while highlighting the relevance of maximal and endurance strength and the relative scarcity of detailed data on anaerobic power, particularly in both upper and lower limbs within this modality [3,10,11]. However, most of the literature has evaluated each sport separately, and combined investigations have primarily examined associations between fitness and simulated combat behavior rather than direct between-sport differences in elite athletes tested under the same protocol [2,4].

This fragmentation limits the extent to which training differences between judo and Brazilian jiu-jitsu can be inferred from the current evidence base and restricts the practical application of these findings for cross-disciplinary training strategies [1,2,3]. Direct cross-sectional comparisons using a common battery of anthropometric, neuromuscular, and grip-related physical performance tests may provide a clearer understanding of whether the shared grappling background of these sports results in similar physical profiles or discipline-specific adaptations [1,4,10]. Therefore, the present study compared the anthropometric profile and physical performance of elite judo and jiu-jitsu athletes. Given the throwing emphasis in judo, which requires rapid concentric force production to project the opponent, and the substantial grip demands shared by both sports, we hypothesized that judo athletes would demonstrate superior lower-limb explosive performance, particularly in tasks emphasizing concentric force, such as the squat jump, while differences in upper-body and grip-related tests were expected to be minimal and were considered exploratory [9,10,12].

## 2. Materials and Methods

### 2.1. Participants

A total of 25 male athletes participated in the study, including 12 judo athletes and 13 jiu-jitsu athletes. All participants had competitive experience at national and international levels, being actively involved in official competitions and representing their clubs in national and international events, with relevant achievements within their respective categories. The athletes were engaged in regular training routines, typically training six days per week, totaling approximately 10 sessions, with an average of two sessions per day. Each training session lasted approximately 2 h, with similar training volume reported between groups. Some athletes reported previous or occasional practice in both judo and Brazilian jiu-jitsu, reflecting the common cross-training context of grappling sports; however, all participants were classified according to their primary competitive modality. Eligibility criteria included (i) being at least 18 years old; (ii) being medically cleared for participation by the club or coaching staff; (iii) being actively involved in competitive preparation; and (iv) having at least two years of structured training and competitive experience in their respective modality. Athletes with musculoskeletal injuries or any condition that could compromise performance during testing were excluded. All participants were informed about the procedures, as well as the potential risks and benefits of the study, and provided written informed consent prior to participation. The study (No. 7.047.156; date of approval: 2 September 2024) was approved by the Research Ethics Committee of the Pontifical Catholic University of Rio Grande do Sul (CEP-PUCRS) and conducted in accordance with the ethical principles of the Declaration of Helsinki.

### 2.2. Design and Setting

This study adopted a cross-sectional design aimed at comparing anthropometric characteristics and physical performance between judo and jiu-jitsu athletes. Participants were recruited through convenience sampling and were assessed during a preparatory phase of their training cycle. Data collection was conducted under standardized conditions. Athletes were instructed to avoid strenuous physical activity for at least 20 h prior to testing, based on practical constraints related to training schedules. All assessments were performed in the morning, starting at approximately 9:00 a.m., around 1 h after breakfast and 90 min after waking, with consistent testing conditions maintained across participants. The evaluation session followed a structured sequence. Initially, anthropometric measurements were collected. Subsequently, participants performed a standardized warm-up consisting of approximately 10 min of dynamic exercises commonly used in their training routines. Following the warm-up, athletes completed the physical test battery described in the subsequent sections.

### 2.3. Procedures

Athletes underwent assessments of anthropometric characteristics and physical performance. The test battery included (i) body mass and stature; (ii) squat jump (SJ); (iii) countermovement jump (CMJ); (iv) medicine ball throw (with and without countermovement); (v) handgrip strength (dominant and non-dominant); and (vi) upper-body strength endurance tests (dynamic and isometric bar). Participants were instructed to maintain their usual dietary habits prior to testing, and all assessments were conducted in a consistent fed state, approximately one hour after breakfast. All assessments were conducted following standardized procedures, with prior familiarization and verbal instructions provided by the evaluators.

#### 2.3.1. Anthropometric and Training Characteristics

Body mass was measured using a digital scale with a resolution of 0.1 kg (G-TECH—Accumed Produtos Médico Hospitalares Ltd.a, Duque de Caxias, Brazil), with athletes barefoot and wearing light clothing. Stature was assessed using a measuring tape fixed vertically to a wall, with a resolution of 1 mm. Athletes were positioned in an upright stance, barefoot, with heels together and head aligned in the Frankfurt plane. Age and training experience (years) were obtained through self-report.

#### 2.3.2. Jump Tests

Participants performed two attempts for each jump condition, with a 30 s interval between attempts and a 2 min interval between the different jump types. The squat jump (SJ) and countermovement jump (CMJ) were performed on a contact mat (Jump System, Cefise, Nova Odessa, Brazil). For the squat jump, athletes started from a standing position with hips and knees flexed at approximately 90°, with the knee angle controlled by visual inspection from experienced evaluators, maintaining their hands on the hips. After an audible signal, the athletes performed a vertical jump through a rapid extension of the lower limbs, without any countermovement. For the countermovement jump, athletes started from an upright standing position with hands on the hips [13]. Following the command, they performed a rapid downward movement (approximately 90° knee flexion), immediately followed by a concentric extension of the lower limbs to execute the jump. Jump height was determined using the Jump System 1.0 software (Jump System, Cefise, Nova Odessa, Brazil), connected to a portable computer, based on flight time. The calculation was performed using the equation h = g × t^2^/8, where h represents jump height, g is the acceleration due to gravity, and t is the flight time [13]. From the two attempts, the highest value was selected for analysis and expressed in centimeters. The reliability of the squat jump was confirmed by an intraclass correlation coefficient (ICC) of 0.951 and a Cronbach’s alpha coefficient of 0.956, while the countermovement jump showed an ICC of 0.954 and a Cronbach’s alpha of 0.954.

#### 2.3.3. Medicine Ball Throw

Participants performed two attempts for each medicine ball throw condition, with a 30 s interval between attempts and a 2 min interval between the different test conditions. Participants were previously familiarized with the test procedures. For the medicine ball throw without countermovement, athletes were positioned in a seated posture with their backs supported against a wall and legs extended in front of the body, holding a 3 kg medicine ball (rubber; circumference: 54–57 cm) commonly used for upper-body power assessment, close to the chest. Upon an audible signal, they were instructed to throw the medicine ball forward with maximal effort, in a straight trajectory, without performing any preparatory movement, with the ball released approximately at eye level to ensure a consistent projection angle and avoid excessive vertical displacement. Participants were instructed to keep their backs in contact with the wall throughout the movement. For the countermovement medicine ball throw, athletes adopted the same initial seated position, with their backs supported against a wall, with the ball held slightly away from the chest [14]. Following the command, they performed a rapid elbow flexion immediately before executing the throw, aiming to maximize performance, maintaining a similar release height to standardize movement execution. The horizontal distance achieved was measured from the wall to the first point of ground contact of the ball. Trials were considered invalid if participants failed to maintain proper body positioning or movement execution, and in such cases, the attempt was repeated to ensure two valid trials. The best result from the two attempts was considered for analysis and expressed in meters. The reliability of the medicine ball throw without countermovement was confirmed by an ICC of 0.927 and a Cronbach’s alpha coefficient of 0.946, while the countermovement condition showed an ICC of 0.960 and a Cronbach’s alpha of 0.961.

#### 2.3.4. Handgrip Strength

Participants performed two attempts for each hand (dominant and non-dominant), in an alternating manner, starting with the dominant hand, as self-reported by the participants [15]. A 30 s interval was allowed between attempts and a 2 min interval between hands. Handgrip strength was assessed using a digital dynamometer (Fitmetria, Cariacica, ES, Brazil; accuracy: 0.1 kg; maximum capacity: 90 kg). The dynamometer grip width was individually adjusted according to each participant’s hand size to ensure optimal force production. Participants performed the test with the elbow flexed at approximately 90°, maintaining the position without external support. Upon an audible command, participants were instructed to perform a maximal isometric contraction, maintaining the effort for approximately 3 s. Verbal encouragement was provided to ensure maximal performance. The highest value obtained for each hand was considered for analysis and expressed in kilograms (kg). The reliability of handgrip strength for the dominant hand showed an ICC of 0.881 and a Cronbach’s alpha of 0.892, while the non-dominant hand presented an ICC of 0.960 and a Cronbach’s alpha of 0.970.

#### 2.3.5. Upper-Body Strength Endurance

Each athlete performed one attempt for each test condition (dynamic and isometric), with a 2 min interval between tests. Both tests were performed using a judogi (kimono) fixed to a horizontal bar, allowing athletes to grip the fabric in a standardized manner [8]. For the dynamic test, athletes started from a suspended position while holding the judogi, with elbows extended, using a self-selected grip width on the fabric, and with bar height adjusted to ensure full body suspension without ground contact. Upon an audible command, they performed repeated pulling actions (elbow flexion and extension) until task failure, aiming to complete the maximum number of correct repetitions possible, following procedures previously described in athletes. The total number of repetitions performed was recorded. For the isometric test, athletes maintained a suspended position holding the judogi, with the elbows flexed (approximately 90°), keeping the chin above the hand level [16]. The objective was to sustain this position for as long as possible. The test was terminated when the athlete was no longer able to maintain the required position. Performance in the dynamic test was expressed as the total number of repetitions, while the isometric test was expressed in seconds, recorded using a digital stopwatch.

### 2.4. Statistical Analysis

Intraclass correlation coefficients (ICCs) and Cronbach’s alpha were used to assess inter-trial reliability in physical performance tests, with the ICC representing relative reliability and Cronbach’s alpha indicating internal consistency across repeated measures. Descriptive statistics are presented as mean and standard deviation for all variables. Data normality and homogeneity were verified using the Shapiro–Wilk and Levene tests, respectively. All variables were tested for normality, with only age and body mass showing non-normal distribution. Comparisons between judo and jiu-jitsu athletes were performed using Student’s *t*-test for independent samples for normally distributed variables, while the Mann–Whitney U test was used for non-normally distributed variables. Effect sizes were calculated using Cohen’s d and interpreted as follows [17,18]: <0.19 (trivial); 0.20–0.49 (small); 0.50–0.79 (moderate); 0.80–1.29 (large); and ≥1.30 (very large). Additionally, an analysis of covariance (ANCOVA) was performed for squat jump performance, using group as a fixed factor and training experience as a covariate, to examine whether the between-group difference remained after adjustment for training experience. The significance level was set at α < 0.05. All analyses were performed using SPSS software (version 22.0; IBM Corp., Armonk, NY, USA).

## 3. Results

Table 1 presents the results of the comparison of anthropometric variables and physical performance between judo and jiu-jitsu athletes. Significant differences were observed only for training experience and squat jump (SJ) performance, both with large effect sizes (ES = 1.472 and ES = 0.850, respectively). Judo athletes showed greater training experience (13.25 ± 2.73 years) compared to jiu-jitsu athletes (7.85 ± 4.36 years) (*p* = 0.001; ES = 1.472), representing a substantial between-group difference. Similarly, in the SJ, judo athletes demonstrated higher performance (38.71 ± 6.69 cm) compared to jiu-jitsu athletes (33.82 ± 4.74 cm) (*p* = 0.045; ES = 0.850). No significant differences were observed between groups for age, height, body mass, handgrip strength (dominant and non-dominant), CMJ, medicine ball throw (with and without countermovement), dynamic pull-up, and isometric pull-up (*p* > 0.05). However, moderate effect sizes were observed for CMJ (ES = 0.554) and MBT-SC (ES = 0.614), despite the absence of statistical significance. An additional ANCOVA performed for SJ, using training experience as a covariate, indicated that the between-group difference was no longer statistically significant after adjustment (F = 1.858; *p* = 0.187; η^2^p = 0.078).

**Figure 1:**
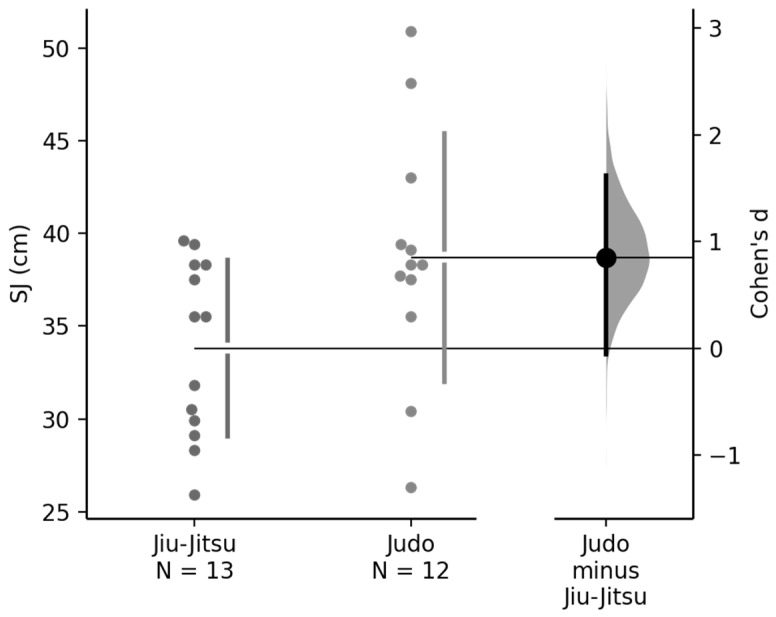
Comparison of squat jump (SJ) performance between judo and jiu-jitsu athletes before adjustment for training experience. The Gardner–Altman plot shows individual values and the mean difference (judo minus jiu-jitsu) with 95% confidence interval. Effect size (Cohen’s d) is also presented.

Figure 1 presents the unadjusted comparison of squat jump (SJ) performance between judo and jiu-jitsu athletes, with the mean difference and confidence interval illustrated in the figure.

## 4. Discussion

In this cross-sectional comparison of elite grapplers, judo athletes displayed greater training experience and higher squat jump performance, whereas no significant between-group differences were observed for age, body size, handgrip strength, countermovement jump, medicine-ball throw, or judogi-grip endurance. These findings suggest a tendency toward similar anthropometric and general physical-performance profiles between elite judo and jiu-jitsu athletes, as no statistically significant differences were detected across most variables, with an initially higher squat jump performance in judo athletes, although this difference was no longer statistically significant after adjustment for training experience.

The higher squat jump performance in the judo group is consistent with evidence that lower-limb explosive strength and power are relevant determinants of judo performance [9,12,19]. Throwing actions require rapid force production against an opponent’s resistance, and higher-level judokas generally exhibit favorable neuromuscular profiles in lower-body power-related measures [7,8,20]. Our data extends this line of evidence by suggesting that this pattern may reflect sport-specific demands, although this interpretation should be considered as a plausible explanation rather than a confirmed mechanism. Moreover, the adjusted analysis suggests that training experience may partially contribute to the initially observed difference in squat jump performance. This finding may partially reflect the specific neuromuscular demands commonly associated with judo performance, although the adjusted analysis indicates that training experience should also be considered when interpreting this result. The absence of a parallel difference in countermovement jump should be interpreted with caution and does not necessarily indicate a selective effect, as it may be influenced by factors such as sample size or measurement variability.

The absence of between-sport differences in handgrip strength, judogi-grip endurance, medicine-ball throw, countermovement jump, and anthropometric measures is also plausible in light of the existing literature [2,3,10,11]. Both sports rely on grip disputes, pulling, isometric stabilization, and repeated upper-body actions, and both have been described as favoring lean, predominantly mesomorphic athletes with substantial strength demands [2,5,10]. Recent evidence further suggests that long-term grappling practice enhances tolerance to severe-intensity handgrip exercise, which may help explain convergent grip-related performance across elite grappling modalities [21]. Additionally, the frequent crossover between judo and Brazilian jiu-jitsu training contexts may contribute to the development of similar neuromuscular and anthropometric characteristics. The fact that the judo group accumulated more years of training without broader superiority across the remaining tests should be interpreted with caution, as training experience represents a relevant confounding variable that should be considered when interpreting between-group differences, particularly because the initially observed difference in squat jump performance was no longer statistically significant after adjustment. This interpretation is also compatible with systematic review evidence showing that strength and power differences are less consistently detected when comparisons are made within highly trained groups than across wider competitive gradients [1].

A second implication of the present results is methodological. Generic physical tests may identify only part of the performance phenotype in grappling sports, especially when athletes are already highly trained [12,22]. In judo, sport-specific classifications such as the Special Judo Fitness Test are related to selected physical qualities, but no single general test appears to define performance comprehensively [12]. In Brazilian jiu-jitsu, the Jiu-Jitsu Anaerobic Performance Test has shown construct validity for discriminating competitive level, again indicating that test specificity matters when the objective is to detect meaningful performance differences [22]. Accordingly, future comparisons between judo and jiu-jitsu should combine general neuromuscular assessments with sport-specific tasks and direct body-composition measures to better isolate truly modality-dependent characteristics [10].

This study has limitations that should temper interpretation. The sample was small and drawn by convenience from a specific competitive context, which reduces statistical precision and external validity, particularly for detecting small-to-moderate between-group differences. The cross-sectional design precludes causal inference, and the greater training experience in the judo group may have contributed to the observed difference in squat jump performance. Importantly, although an adjusted analysis was carried out, the substantial difference in training experience should still be considered when interpreting the present findings, particularly given the small sample size and the cross-sectional design. Therefore, the present findings should be interpreted with caution when generalizing to other populations or competitive levels. Future studies should recruit larger multicenter samples, stratify by sex and weight category, include body-composition measurements and sport-specific performance tests, and preferentially apply longitudinal designs capable of tracking how training history and competitive exposure shape discipline-specific adaptations.

From an applied perspective, the present findings support a common conditioning foundation for elite grapplers, particularly for grip capacity, upper-body endurance, and general strength-power maintenance [1,4]. However, judo programs may consider emphasizing concentric lower-limb explosiveness, given the sport’s throwing demands and the initially higher squat jump values observed in judo athletes [9,12]. Conversely, the similarity across most other tests suggests that discipline-specific technical preparation may be more discriminative than broad fitness batteries when athletes are already operating at an elite level. A key strength of this study is the inclusion of elite-level athletes assessed under standardized conditions, which enhances the relevance of the findings for high-performance settings.

## 5. Conclusions

Among elite grappling athletes, judo competitors showed greater training experience and superior squat jump performance, although the substantial difference in training experience between groups should be considered when interpreting this result, whereas the remaining anthropometric and physical-performance measures were broadly similar between judo and Brazilian jiu-jitsu. These findings suggest a tendency toward similar general physical profiles at the elite level, as no statistically significant differences were detected across most variables, while the initially observed difference in squat jump performance was no longer statistically significant after adjustment for training experience. From an applied perspective, these results highlight that conditioning programs in both sports may rely on similar general physical foundations, although judo athletes initially demonstrated higher squat jump values. The study adds direct comparative evidence to the literature, which has mostly examined the two sports separately, and reinforces the importance of incorporating sport-specific and context-sensitive assessments in future comparative research.

## Figures and Tables

**Table 1:** Comparison and effect size of anthropometric characteristics and physical performance between judo and jiu-jitsu athletes.

				95% Confidence Interval			
	Group	Mean	Standard Deviation	Lower Limit	Upper Limit	*p*	ES
Age (years)	Judo	19.58	1.62	18.55	20.61	0.079	0.410
	Jiu-Jitsu	22.77	5.04	19.73	25.81		
TE (years)	Judo	13.25	2.73	11.51	14.99	0.001 *	1.472
	Jiu-Jitsu	7.85	4.36	5.21	10.48		
Height (cm)	Judo	177.67	7.33	173.01	182.32	0.905	−0.048
	Jiu-Jitsu	178.08	9.39	172.40	183.75		
BM (kg)	Judo	85.16	20.55	72.11	98.22	0.493	0.279
	Jiu-Jitsu	79.87	17.43	69.34	90.40		
HGS-D (kg)	Judo	53.35	9.98	47.01	59.69	0.659	−0.179
	Jiu-Jitsu	55.19	10.59	48.79	61.59		
HGS-ND (kg)	Judo	51.40	10.83	44.52	58.28	0.888	−0.057
	Jiu-Jitsu	52.05	11.72	44.96	59.13		
SJ (cm)	Judo	38.71	6.69	34.46	42.96	0.045 *	0.850
	Jiu-Jitsu	33.82	4.74	30.95	36.68		
CMJ (cm)	Judo	40.82	7.45	36.08	45.55	0.180	0.554
	Jiu-Jitsu	37.25	5.34	34.03	40.48		
MBT-SC (m)	Judo	5.30	0.72	4.85	5.75	0.139	0.614
	Jiu-Jitsu	4.85	0.74	4.41	5.30		
MBT-CC (m)	Judo	5.55	0.76	5.07	6.03	0.310	0.416
	Jiu-Jitsu	5.21	0.88	4.68	5.74		
DPU (reps)	Judo	17.33	7.46	12.59	22.08	0.210	−0.516
	Jiu-Jitsu	21.15	7.36	16.71	25.60		
IPU (s)	Judo	51.30	16.24	40.98	61.62	0.772	−0.117
	Jiu-Jitsu	53.46	20.19	41.26	65.66		

Abbreviations: TE = training experience; BM = body mass; HGS = handgrip strength; D = dominant; ND = non-dominant; SJ = squat jump; CMJ = countermovement jump; MBT = medicine ball throw; SC = without countermovement; CC = with countermovement; DPU = dynamic pull-up; IPU = isometric pull-up; ES = effect size; * significant difference between groups (*p* < 0.05).

## Data Availability

The data presented in this study are available upon reasonable request from the corresponding author. The data are not publicly available due to privacy and ethical restrictions related to the potential identification of the elite athletes included in the study.
